# Livestock-Associated MRSA in Household Members of Pig Farmers: Transmission and Dynamics of Carriage, A Prospective Cohort Study

**DOI:** 10.1371/journal.pone.0127190

**Published:** 2015-05-18

**Authors:** Brigitte A. G. L. van Cleef, Birgit H. B. van Benthem, Erwin J. M. Verkade, Miranda M. L. van Rijen, Marjolein F. Q. Kluytmans-van den Bergh, Haitske Graveland, Thijs Bosch, Koen M. H. W. Verstappen, Jaap A. Wagenaar, Marian E. H. Bos, Dick Heederik, Jan A. J. W. Kluytmans

**Affiliations:** 1 Laboratory for Microbiology and Infection Control, Amphia Hospital, Breda, the Netherlands; 2 Laboratory for Medical Microbiology and Immunology, St. Elisabeth Hospital, Tilburg, the Netherlands; 3 Centre for Infectious Disease Control Netherlands, National Institute for Public Health and the Environment, Bilthoven, the Netherlands; 4 Amphia Academy Infectious Disease Foundation, Amphia Hospital, Breda, the Netherlands; 5 Julius Center for Health Sciences and Primary Care, UMC Utrecht, Utrecht, the Netherlands; 6 Institute for Risk Assessment Sciences, Utrecht University, Utrecht, the Netherlands; 7 Department of Infectious Diseases and Immunology, Utrecht University, Utrecht, the Netherlands; 8 Central Veterinary Institute of Wageningen UR, Lelystad, the Netherlands; 9 Department of Medical Microbiology and Infection prevention, VU University medical center, Amsterdam, the Netherlands; Ross University School of Veterinary Medicine, SAINT KITTS AND NEVIS

## Abstract

This prospective cohort study describes carriage of livestock-associated methicillin-resistant *Staphylococcus aureus* (LA-MRSA) in household members from 49 farrowing pig farms in the Netherlands (2010–2011). Of 171 household members, 4% were persistent MRSA nasal carriers, and the MRSA prevalence on any given sampling moment was 10% (range 7-11%). Working in the stables (of which 98% was MRSA-positive, prevalence ratio (PR) = 2.11 per 10 hours), working with sows (PR=1.97), and living with an MRSA-positive pig farmer (PR=4.63) were significant determinants for MRSA carriage. Significant protective factors were carriage of methicillin-susceptible *Staphylococcus aureus* (MSSA) (PR=0.50), and wearing a facemask when working in the stables (37% decreased prevalence). All MRSA strains during the study period were known livestock-associated types. The bacteriophage *φ*3 was not found in household members. Transmission from pigs and the environment appeared to be important determinants; human-to-human transmission could not sufficiently be differentiated. Wearing a facemask when working in the stables and carriage of MSSA are potential interventional targets.

## Introduction

Livestock-associated methicillin-resistant *Staphylococcus aureus* (LA-MRSA, multi locus sequence type 398) is a relatively new MRSA clade (*i*.*e*. subtype coming from one ancestor), first described in 2005 [1]. The prevalence of LA-MRSA carriage in people working with livestock animals ranges from 20 to 63% [2–8], the prevalence in pigs and veal calves rises up to 75% [9]. Direct contact with livestock was shown to be a major risk factor for carriage of LA-MRSA [4, 5, 8].

In household members of livestock farmers, the prevalence varies from 4 to 16% [4, 10, 11]. Their determinants of carriage, as well as the exact roles of human-to-human transmission and the home environment are yet undetermined. Therefore, this study aims to describe the dynamics of carriage of LA-MRSA in household members of pig farmers. The results of this study provide targets for limiting the acquisition and spread of LA-MRSA.

## Materials and Methods

### Study design and selection of farms

This prospective cohort study surveyed persons living on 49 farrowing pig farms in the Netherlands for 1 year (2010–2011). Pig farms were randomly selected among participants from a previous study [9], which contained randomly selected farrowing pig farms from all Dutch pig farms. A detailed analysis of determinants of MRSA and methicillin-susceptible *S*. *aureus* (MSSA) carriage in the pig farmers of this study has been described elsewhere [8].

### Sampling moments

During the 1-year study period, there were six sampling moments: day 0, day 4, day 7, month 4, month 8, and month 12. On day 0, quantitative nasal and oropharyngeal Eswabs (Copan, Brescia, Italy), and extensive questionnaires, regarding exact activities on the pig farm, contact with animals, hospital contact, personal use of antimicrobials or immunosuppressive drugs, underlying disorders (e.g. eczema or other skin diseases) and presence of indwelling catheters and/or open wounds, were collected. Wet wipe samples (Sodibox, Nevez, France) of four defined surfaces in house (backdoor handle, television remote control, chair of pig farmer, and dog neck/back) and four surfaces in the stables (farrowing and weaning stables, in duplicate) were collected as well. Nasal swabs were introduced in the nostril and rotated once. Oropharyngeal swabs sampled the area of the inner cheek including the tonsils. Refrigerated swabs were transported to the laboratory, and cultured within 24 hours. In addition, dry electrostatic dust collector cloths (EDCs) [12] were placed in the farrowing and weaning stables (in duplicate) and on the highest cupboard in the living room of the house, and were left in place for 2 weeks before quantitative analysis.

On the remaining sampling moments, nose dry swab (Copan) self-samples were analysed semi-quantitatively and short questionnaires were filled in. Swab instructions were sent with the swabs. EDCs were placed on the same five locations in months 4, 8 and 12. An additional throat self-sample was analysed semi-quantitatively in month 12. Results of the individual cultures were disclosed at the end of the study.

### Definitions

Household members were defined as individuals who lived on the pig farm premises, and worked in the stables for less than 20 hours per week at the start of the study. Pig farmers were defined as individuals who lived on the pig farm premises, and worked in the stables for at least 20 hours per week at the start of the study.

Persistent carriers were defined as persons with all nasal cultures positive for MRSA, regardless of typing results, non-carriers had no positive cultures, and intermittent carriers were the remaining persons.

### Laboratory analysis: cultures

The extended laboratory procedure is described elsewhere [8]. In short, quantitative cultures were performed by serially diluting ESwab—medium and incubating on chromID *S*. *aureus* and chromID MRSA agar plates (BioMérieux, La Balme Les Grottes, France). The number of CFU was counted on both plates. Semi-quantitative cultures were performed with dry swabs on the same media, directly plated and after enrichment. Wet wipe samples were enriched in two consecutive media and subsequently cultured on blood and *Brilliance* MRSA agar plates (Oxoid, Basingstoke, UK).

All *S*. *aureus* strains were defined by green colonies on selective *S*. *aureus* agar in combination with a positive coagulase slide and DNase test. Methicillin susceptibility was tested using the cefoxitin disk diffusion method according to EUCAST standards [13], followed by a duplex PCR for the *nuc* and *mec*A genes as described previously [14].

### Laboratory analysis: EDC PCR

For each EDC sample, four targets were detected with a LightCycler 480-II real-time quantitative PCR (Roche Diagnostics, Almere, the Netherlands): (i) *mec*A for methicillin-resistance [15], (ii) C01 for sequence type 398 [16], (iii) *fem*A [15] and (iv) *nuc* [17] for detection of *S*. *aureus* [8].

The numbers of CFU-equivalents (eqCFU) per PCR and per EDC were calculated. The *S*. *aureus* concentration was the maximum of either *fem*A or *nuc*; the concentration of MRSA was the minimum of *mec*A and *S*. *aureus*; the concentration of LA-MRSA was the minimum of MRSA and C01.

### Molecular typing

MRSA isolates from nares, oropharynx and wet wipes were genotyped with staphylococcal protein A (*spa*) typing and multiple-locus variable number of tandem repeat analysis (MLVA), as described previously [18, 19]. All MRSA strains from pig farmers, household members, employees and surface samples were tested for the presence of the bacteriophage *φ*3, a possible marker of increased human-to-human transmissibility [20, 21], with an in-house PCR method.

### Statistical analysis

An extended description of statistical analysis can be found elsewhere [8]. In short, data were analysed with SAS, version 9.3 (SAS Institute Inc., Cary, NC, USA). For each person, the proportion of nasal cultures positive for MRSA, methicillin-susceptible *S*. *aureus* (MSSA) and *S*. *aureus* in general was calculated, resulting in persistent, intermittent and non-carriers. The effect of exclusive MSSA nasal carriage on MRSA carriage was studied in sets of two consecutive sampling moments in persons initially without MRSA.

Univariate and multivariate calculations used PROC GENMOD, a generalized estimated equations model, with nasal MRSA carriage in household members as dependent variable (persistent and intermittent carriage combined versus non-carriage). All determinants with univariate Chi-square p-values of ≤0.20, a prevalence in household members of >5%, and a number of missing observations ≤20% were eligible for multivariate analysis. When Spearman’s rho for two determinants was >0.70, colinearity was assumed, and the determinant with the highest prevalence ratio (PR) [22] or lowest p-value was selected for the multivariate analysis.

### Ethical considerations

All subjects provided written informed consent before entering the study. The study protocol was approved by the medical ethical committee of the St Elisabeth Hospital in Tilburg, the Netherlands (protocol number 0933).

## Results

### MRSA carriage

**Fig 1:**
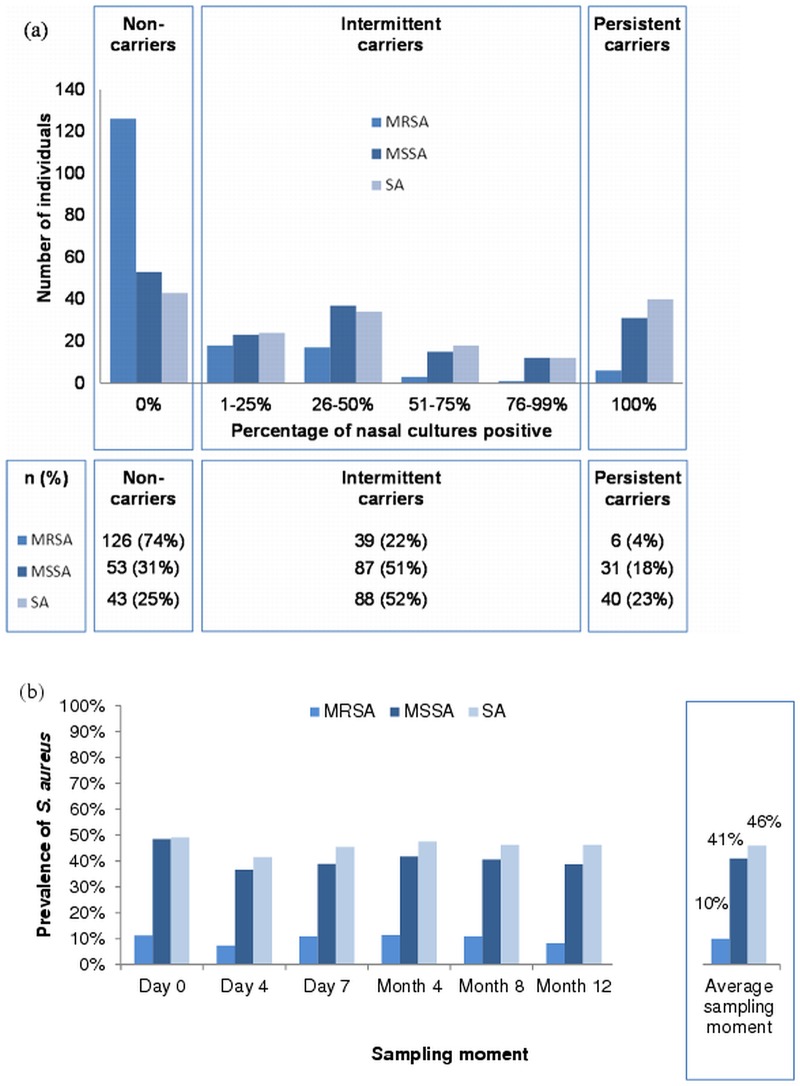
Carriage^a^ of MRSA, MSSA and *S*. *aureus* ^b^ in household members of pig farmers (a) and mean cross-sectional nasal MRSA, MSSA and *S*. *aureus* ^b^ prevalences per sampling moment (b). ^a^ A persistent carrier was a person with all nasal cultures positive, non-carriers had no positive cultures, intermittent carriers were the remaining persons. ^b^ Since MRSA and MSSA could co-exist in one sample, and *S*. *aureus* carriage could be a combination of MRSA and/or MSSA, the numbers do not add up. For example, a person carried MRSA on four out of six sampling moments, and MSSA on the remaining two sampling moments. This person was an intermittent MRSA carrier, an intermittent MSSA carrier, but a persistent *S*. *aureus* carrier.

This 1-year prospective cohort study included 171 household members from 45 pig farms (68 men), with a median age of 16 years (range 0–70). Four farms, in which there were no household members apart from the farmer, were excluded from the analysis. The median number of working hours per week in the stables at the start of the study was 1 (range 0–18). Fig 1a demonstrates that 4% of household members were persistent MRSA nasal carriers. MRSA was found at least once in 23% of the partners of farmers (6/26), 19% of the children (20/105) and both parents of farmers (2/2) during the study period. Variation in MRSA prevalence and cross-sectional prevalence per sampling moment are shown in Fig 1b, the average MRSA prevalence was 10% (range 7–11%). Inclusion of oropharyngeal samples in the definition of persistence resulted in 67% non-carriage, 29% intermittent carriage and 4% persistent carriage (74%– 22%– 4% without oropharyngeal samples, respectively, Fig 1a). Out of 1021 samples, 49 were missing (5%). Sensitivity analysis, defining the missing samples as either MRSA positive or negative, did not reveal much variance in carriage rates.

**Fig 2:**
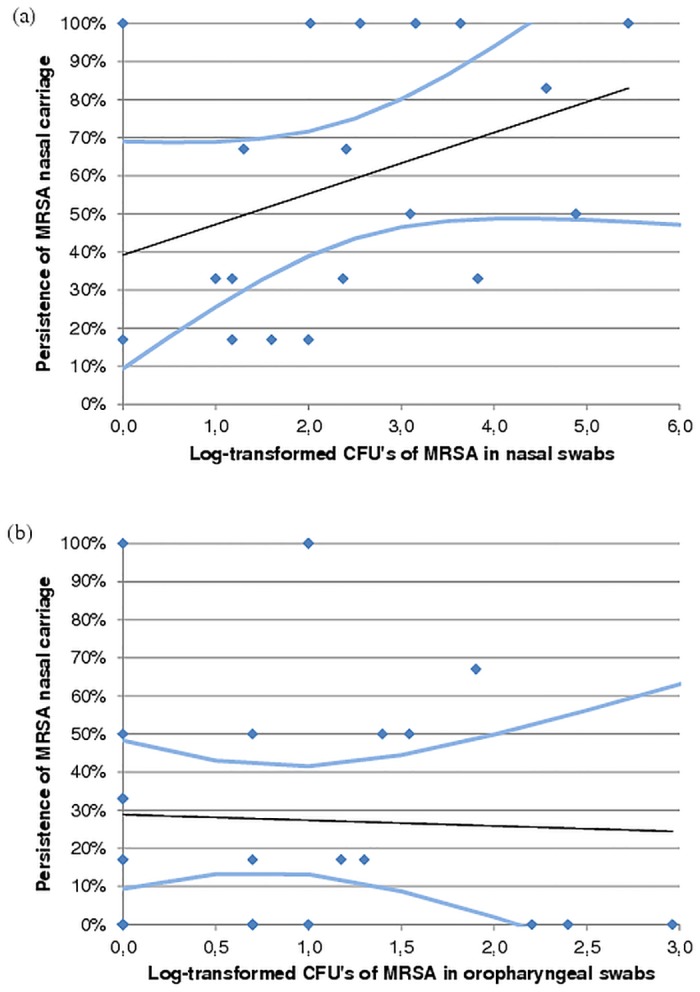
Linear regression model and 95% confidence bands between log-transformed colony forming units of methicillin-resistant *Staphylococcus aureus* in nasal (A) and oropharyngeal (B) swabs from start of study (x-axis) and persistence of MRSA nasal carriage during one year (y-axis) in household members who were MRSA-positive at start of the study. Prevalence ratio (PR) per log CFU = 1.23, 95% confidence interval 0.95–1.60, p = 0.11 for nasal samples. PR = 0.78, 95% CI 0.33–1.88, p = 0.58 for oropharyngeal samples.

The median number of MRSA in nasal samples at the start of the study was 2.4 log-transformed CFU, interquartile range (IQR: p25-p75) = 1.2–3.6), regarding positive samples only. For oropharyngeal samples a median of 0.7 log CFU (IQR = 0.0–1.3) was found. The presence of MRSA in oropharyngeal swabs at the start of the study was statistically significantly associated with MRSA nasal carriage (PR = 2.47, 95% 95% confidence interval (CI) 1.44–4.24, p = 0.00). Within the group of MRSA carriers at the start of the study, household members with a higher number of CFU showed a trend of being more often a persistent nasal carrier, as shown in Fig 2.

Pig farmers carrying MRSA persistently were present in 22 pig farms, in which 31% of the household members carried MRSA during the study (persistently or intermittently, 29/94). Pig farmers carrying MRSA intermittently originated from 3 farms, in which 30% of household members MRSA were positive (14/46). Non-carrying pig farmers came from 10 farms, in which 6% of household members were MRSA positive (2/31). MRSA positive pig farmers and household members (persistent or intermittent) often coincided (PR in multivariate analysis 4.63, p = 0.02, Table 1).

**Table 1:** Determinants of MRSA nasal carriage in household members of pig farmers after multivariate analysis.

Determinant	PR (95% CI)	p-value
Working in stables—per 10 hours/week	2.11 (1.56–2.85)	**<0.0001**
Taking care of sows in the last 7 days	1.97 (1.26–3.08)	**<0.0001**
Exclusive MSSA at start study	0.50 (0.28–0.88)	**0.02**
Member of family with MRSA-positive pig farmer	4.63 (1.23–17.38)	**0.02**

MRSA, methicillin-resistant *Staphylococcus aureus*; MSSA, methicillin-susceptible *Staphylococcus aureus*; PR (95% CI), Prevalence ratio with 95% confidence intervals. Bold-typed p-values were statistically significant (i.e. <0.05).

### *S*. *aureus* versus MRSA

As shown in Fig 1, the prevalence of persistent nasal *S*. *aureus* carriage (MRSA and MSSA combined) in household members was 23%, and the prevalence of *S*. *aureus* carriage at the different time points was on average 46%.

Exclusive MSSA nasal carriage (without MRSA) was negatively associated with the acquisition of MRSA as measured on the next sampling moment, although only when those moments were months apart (day 0, month 4, month 8, month 12), as shown in Table 2. When considering sampling moments that were days apart, no significant association was seen.

**Table 2:** Effect of exclusive MSSA nasal carriage on MRSA carriage in the next sampling moment.

	Previous MSSA carriage—no. (%)^a^		PR (95% CI)	p-value
	Yes	No		
MRSA carriers, high frequency sampling^b^	5 (4)	8 (4)	0.96 (0.32–2.84)	0.94
MRSA carriers, low frequency sampling^c^	2 (1)	17 (7)	0.17 (0.04–0.74)	**0.02**
MRSA carriers, all sampling moments^d^	10 (4)	25 (6)	0.63 (0.31–1.26)	0.19

MRSA, methicillin-resistant *Staphylococcus aureus*; MSSA, methicillin-susceptible *Staphylococcus aureus*; PR (95% CI), Prevalence ratio with 95% confidence intervals. Bold-typed p-values were statistically significant (i.e. <0.05). Since only MRSA carriers were considered in this table, numbers were low.

^a^ Only persons at risk for MRSA acquisition were considered, i.e. MRSA negative on the previous sampling moment.

^b^ High frequency sampling moments: day 0, day 4, day 7.

^c^ Low frequency sampling moments: day 0, month 4, month 8, month 12.

^d^ All sampling moments: day 0, day 4, day 7, month 4, month 8, month 12. Numbers do not add up, since day 0 was included in both subgroups, resulting in different sets of samples.

### Environmental samples

At the start of the study, 82% of wet wipe samples and 98% of dry EDCs in the stables were MRSA-positive (Table 3). MRSA carriage was significantly associated with the presence of MRSA in dog wipe samples, for other wet wipe samples no association was found. MRSA in dry EDC samples from the home environment was univariately associated with MRSA in household members; in multivariate analysis no significant association was seen (S1 Table).

**Table 3:** Environmental samples positive for MRSA.

	All farms^a^ (n = 45)	Farms with household members who were^b^		p-value^c^
		MRSA carriers^b^ (21 farms)	MRSA negative^b^ (24 farms)	
*Wet wipe samples at start of study—no*. *positive farms/total no*. *farms (%)*				
Stables	37/45 (82)	19/21 (90)	18/24 (75)	0.25
House	28/44 (64)	15/21 (71)	13/23 (57)	0.31
Door handle	12/44 (27)	8/21 (38)	4/23 (17)	0.12
Remote control	11/44 (25)	6/21 (29)	5/23 (22)	0.60
Chair	26/43 (60)	13/20 (65)	13/23 (57)	0.57
Dog	11/33 (33)	8/16 (50)	3/17 (18)	**0.05**
*EDCs at start of study—no*. *positive farms/total no*. *farms (%)*				
Stables	44/45 (98)	20/21 (95)	24/24 (100)	0.47
House	3/44 (7)	3/21 (14)	0/23 (0)	0.10
*EDCs during study—median LA-MRSA eqCFU per cloth (IQR)*				
Stables		160 (56–460)	92 (39–233)	**0.03**
House		0 (0–0)	0 (0–0)	0.13

MRSA, methicillin-resistant *Staphylococcus aureus*; MSSA, methicillin-susceptible *Staphylococcus aureus*; PR (95% CI), Prevalence ratio with 95% confidence intervals; IQR, interquartile range (p25–p75); eqCFU, colony forming units equivalents.

^a^ Four farms did not have household members.

^b^ MRSA carriers were carrying MRSA nasally persistently or intermittently, MRSA negative persons did not have any MRSA during the study.

^c^ P-values between proportions were calculated with Chi-square tests or Fisher’s Exact tests when 50% of the expected cell values were <5, EDC eqCFU counts were compared using Wilcoxon-Rank-Sum tests. Bold typed numbers indicate significance.

Pig farms with MRSA-positive household members had significantly higher median stable-EDC LA-MRSA concentrations, compared to pig farms with no MRSA-positive household members (Table 3).

### Determinants for MRSA carriage

Univariate determinants of MRSA carriage in household members (persistent and intermittent carriage combined) are depicted in S1 Table. Age, number of hours per week working in the stables, exclusive MSSA carriage at start of the study, contact with sheep, various tasks in the stables (among which giving antimicrobials to pigs, working with sows, piglets, weaned piglets, and finisher pigs), and presence of MRSA in wet wipe samples of remote control and the farmers chair were significantly associated with MRSA carriage in univariate analysis.

Continuously wearing a facemask when working in the stables and less contact with pigs in the last year were determinants with >20% of missing observations, but significantly negatively associated with MRSA carriage (S1 Table). Household members continuously wearing a facemask when working in the stables had a 37% decreased prevalence of MRSA carriage during the study (PR for non-carriage 1.37, 95% CI 1.11–1.70, p<0.0001). Sensitivity analysis, i.e. placing the missing samples in either category of wearing face masks, did not reveal a different PR or significance level. In addition, wearing a face mask was not associated with specific farm tasks, or farm management (e.g. hygiene regulations, size of farm).

The number of hours per week working in the stables, taking care of sows, and being a household member of an MRSA-positive pig farmer was significantly associated with the risk of MRSA carriage during the study in multivariate analysis (Table 1). Exclusive carriage of MSSA (without MRSA) at the start of the study was significantly associated with a 50% decreased risk of MRSA carriage on any later sampling moment. Excluding the number of hours per week working in the stables from the analysis did not result in a different model.

### Molecular typing

In total, 110 MRSA strains from 47 household members were typed using MLVA (S1 Fig). All MRSA isolates from nose, oropharynx and wet wipe samples had *spa*-types and MLVA-types (MTs) corresponding to MLVA complex (MC) 398, also known as the livestock-associated clade [23]. Ninety percent of MRSA isolates from household members carried MLVA-types MT572 (n = 40 isolates), MT398 (n = 38), or MT567 (n = 21). *Spa*-typing showed that 89% of MRSA isolates belonged to *spa*-types t108 (n = 40), t011 (n = 36), t899 (MT567, n = 12), or t034 (MT569 and MT556, n = 10), where *spa*-type t011 generally matched with MT398 and *spa*-type t108 with MT572. The remaining *spa*-types found were t1255 (MT567, n = 8), t3479 (MT398, n = 2), t1184 (MT567, n = 1), and t4852 (MT572, n = 1).

Five out of six household members (83%) who were carrying MRSA persistently carried the same MT or a single-locus variant (SLV) throughout the study. This group of persons consisted of three persons that carried MT398 consistently, one person carried MT572 and one person carried a combination of MT398 and MT572. In addition, MRSA strains from all eight persons who were positive in both nose and oropharynx at the same sampling moment yielded the same MT or a SLV. In 21 farms, both pig farmers and their household members carried MRSA during the study. Within 6 of these 21 farms (29%) all persons had LA-MRSA with similar MT or a SLV.

The bacteriophage φ3 was found in 3% (20/794) of all MRSA isolates in this study. These 20 isolates were obtained from three pig farmers, three employees (both groups sometimes carrying the bacteriophage φ3 on multiple moments) and four surface samples, originating from six farms. All MRSA isolates with the bacteriophage φ3 were of MT398 and *spa*-type t011. None of the household members carried MRSA isolates with the bacteriophage φ3.

## Discussion

### Dynamics of *S*. *aureus* carriage in household members of pig farmers

MRSA was present in 26% of household members (4% persistent carriage, 22% intermittent carriage) during the study, with a mean prevalence of 10% on any given sampling moment. The first sampling moment showed a higher prevalence, which can probably be explained by the additional quantitative measurements performed at that moment only. The mean prevalence is somewhat higher than generally reported in household members of pig or veal farmers in the literature, being 4–8% [4, 5, 10]. However, Graveland et al. recently described MRSA in 53% of 97 household members of veal farmers (2% persistent carriage, 51% intermittent carriage), with a mean prevalence of 16% [11]. The more intense sampling in the latter study (on average 10 swabs in two months) might have resulted in a lower number of persistent carriers (the more samples, the higher chance for a negative one, resulting in less persistence), and a higher number of intermittent carriers, compared to our study.

*S*. *aureus* (MRSA and MSSA combined) carriage rates from this study (23% persistent carriage, 52% intermittent carriage, 25% non-carriage) were comparable to those found in literature [24, 25]. Non-carriers were more prevalent in literature, which is possibly a result of the extensive exposure to *S*. *aureus* in the stables, homes and pig farmers of the specific population studied here. MRSA carriage does not seem to change *S*. *aureus* numbers much (Fig 1), only non-carriage falls from 31% (MSSA) to 25% (SA), a relative difference of 19%. Whether this is because of addition or replacement cannot be concluded from this study. The detection of MSSA in the presence of MRSA in the same sample may be affected as well (see Study limitations).

### Contact with pigs

Primary exposure to MRSA-positive pigs appeared to be an important risk factor for nasal MRSA carriage of household members during the study. This was demonstrated by the finding that working in stables and working with sows were significant determinants in the multivariate analysis, the observed association with the frequency of pig contact in univariate analysis, and the extreme exposure likelihood in the stables (82% of wet wipe samples and 98% of EDCs were MRSA-positive). The finding that pig contact is an important determinant of nasal MRSA carriage is confirmed by the literature [4, 10, 11, 26, 27].

In addition, household members continuously wearing facemasks when working in the stables had 37% less MRSA carriage, compared to household members who sometimes or never wore a facemask when working in the stables, confirming that the risk is coming from the presence of MRSA in the stables. The use of facemasks to prevent acquisition of microbes is common practice in healthcare. In the Netherlands, it is recommended to wear a facemask when working in the stables to prevent acquisition of MRSA by healthcare workers when caring for MRSA-positive patients [28]. However, there are few studies regarding the effectiveness of facemasks for prevention of acquisition of MRSA or other micro-organisms [29]. Pig farmers are generally advised to wear them because of the heavy environmental dust load and bacterial exposure in the stables [30]. This observational study showed a protective effect in both household members and pig farmers [8], which should be confirmed in a prospective trial as it offers a simple, cheap and effective measure to prevent occupational acquisition of MRSA.

### Contact with MRSA-positive pig farmers and MRSA in the environment

The presence of an MRSA-positive pig farmer was found to be significantly associated with MRSA carriage in household members in multivariate analysis. However, it is not possible to distinguish between human-to-human transmission and transmission via the stables or home environment in this study. Almost all household members visit the stables at least occasionally, resulting in exposure to MRSA-positive pigs and/or dust. Alternatively, the pig farmer will very likely contaminate home surfaces directly with MRSA from the stables, or with own colonizing MRSA strains, and MRSA can transfer to household members from these surfaces.

A higher MRSA load present in the environment was associated with MRSA carriage in household members, suggesting a large role for environmental contamination, as described before [31, 32]. Only 29% of farms with MRSA-positive persons had MLVA types comparable between all persons in a farm. Lastly, the bacteriophage *φ*3, carrying the virulence genes *chp* and *scn*, and thought to be the best genetic marker for human-to-human transmission of LA-MRSA since it is specifically found in human-associated clades [20, 21], was not present in LA-MRSA isolates of household members from this study. Therefore, the environment appears to be a very important determinant for human carriage, and true human-to-human transmission rates are better studied in other settings. In a study where household members of pig veterinarians did not have contact with livestock, transmissibility of LA-MRSA appeared to be reduced, compared to non-LA-MRSA [33].

### MSSA is negatively associated with MRSA

In multivariate analysis, MSSA nasal carriage in household members was associated with absence of MRSA during the study. This association is confirmed regarding consecutive sets of samples, where exclusive MSSA nasal carriage (without MRSA) was associated with a 83% decreased risk of MRSA acquisition on the next sampling moment, but only when those moments are months apart. The nasal presence or absence of MRSA appears to vary with a frequency of months, instead of days. More studies have demonstrated the existence of bacterial competition, not only for *Staphylococcal* spp. among themselves, but also between genera [34, 35]. The present study in pig farmers [8], and a recent study in veal calf farmers found a negative association between MSSA and MRSA carriage as well [11]. This may offer an opportunity for interventions to reduce the rates of MRSA in livestock. Specific protecting MSSA-types can be further studied in future as well, since in this study only MRSA strains were typed.

### Study limitations

The samples from this study were largely based on self-sampling techniques, which might not have been sufficient, resulting in a possible underestimation of MRSA carriage. However, a pilot study showed excellent agreement for the nose (agreement = 93%, kappa = 0.85, 95% CI 0.74–0.96) and good agreement for oropharyngeal samples (agreement = 83%, kappa = 0.60, 95% CI 0.43–0.76) [36]. Moreover, the high MRSA carriage rates found in this study, compared to other studies, do not indicate an underestimation.

Furthermore, the culture method is limited in detecting MSSA and MRSA in one sample as different entities. When a sample was MRSA-positive, the potential presence of MSSA in the same sample might have been missed, because only when two morphologically different colonies existed on the blood agar plate, both were determined. In that case, the number of MSSA CFU was the number of SA CFU minus the number of MRSA CFU. The reported numbers of MRSA, isolated MSSA, or *S*. *aureus* carriage in general are correct, because only samples with simultaneous presence of MRSA and MSSA are affected. In addition, the negative association found between MRSA acquisition and exclusive MSSA carriage is correct, since this was studied in MRSA-negative samples where underestimation of MSSA was not an issue. Therefore, we believe that this effect is of negligible impact on our results.

Third, some potential risk factors had >20% missing observations, leading to exclusion from multivariate analysis. This limits the number of variables that could be studied but not the reliability of the variables that were included in the analysis.

## Conclusions and Recommendations

We conclude that pig contact and transmission from the environment are important determinants of MRSA carriage in household members of pig farmers. Human-to-human transmission needs more research. MSSA carriage and wearing facemasks when working in the stables may offer possible interventions for the acquisition of MRSA in this highly endemic setting.

## Supporting Information

S1 Fig Minimum spanning tree of MLVA-results of methicillin-resistant *Staphylococcus aureus* isolates of household members, pig farmers, employees and wet wipe samples. Each circle represents a MLVA-type with the name of the larger groups printed next to the circle, and the size of the circle symbolizes the amount of isolates belonging to this type.
(TIF)

S1 Table Determinants of MRSA nasal carriage in household members of pig farmers, univariate analysis^a^. (DOCX)
