# Supplementary figures and images for: Livestock-Associated MRSA in Household Members of Pig Farmers: Transmission and Dynamics of Carriage, A Prospective Cohort Study
Source: PLoS One. 2015 May 18;10(5):e0127190. doi: 10.1371/journal.pone.0127190 (PMC4436301; doi:10.1371/journal.pone.0127190)

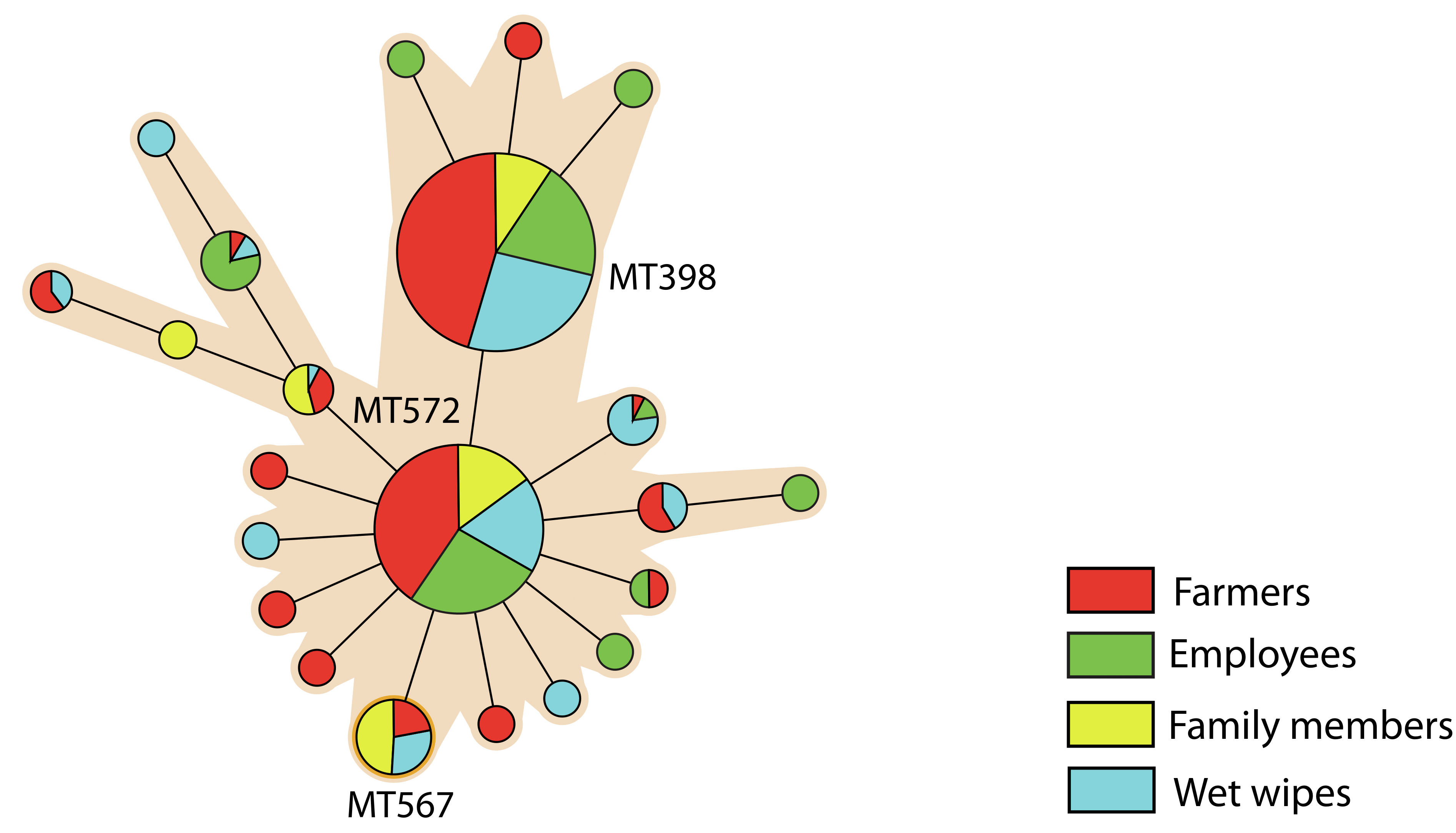

Supplement: S1 Fig — Each circle represents a MLVA-type with the name of the larger groups printed next to the circle, and the size of the circle symbolizes the amount of isolates belonging to this type. (TIF) [file pone.0127190.s001.tif]
