# Supplementary material for: Livestock-Associated MRSA in Household Members of Pig Farmers: Transmission and Dynamics of Carriage, A Prospective Cohort Study
Source: PLoS One. 2015 May 18;10(5):e0127190. doi: 10.1371/journal.pone.0127190 (PMC4436301; doi:10.1371/journal.pone.0127190)
Supplement: S1 Table — (DOCX) [file pone.0127190.s002.docx]

**Supplementary table S1**

**Table S1. Determinants of MRSA nasal carriage in household members of pig farmers, univariate analysis^a^.**

| **Determinant** | | **Value** | **Total -  no. (n=171)** | **MRSA -  no. (%) (n=45)** | **p-value** |
| --- | --- | --- | --- | --- | --- |
| *General determinants* | |  |  |  |  |
| Age - per 10 years | |  |  |  | **0.01** |
| Gender | | Male Female Missing | 68 102 1 | 22 (32) 23 (23) 0 (0) | 0.20 Ref |
| Exclusive MSSA at the start of the study | | Yes No Missing | 67 102 2 | 8 (12) 36 (35) 0 (0) | **<0.0001**  Ref |
| *Contact with animals* | |  |  |  |  |
| Contact with sheep in the last 12 months | | Yes No Missing | 16 152 3 | 6 (38) 38 (25) 0 (0) | **0.08** Ref |
| Contact with poultry in the last 12 months | | Yes No Missing | 17 146 8 | 6 (35) 37 (25) 0 (0) | 0.14 Ref |
| *Working in the stables* | |  |  |  |  |
| Worked in stables - per 10 hours/week | |  |  |  | **<0.0001** |
| Gave antimicrobials to pigs in the last 7 days | | Yes No Missing | 15 150 6 | 9 (60) 34 (23) 0 (0) | **<0.0001** Ref |
| Health care of sows in the last 7 days | | Yes No Missing | 20 148 3 | 12 (60) 32 (22) 0 (0) | **<0.0001** Ref |
| Birth assistance of sows in the last 7 days | | Yes No Missing | 18 150 3 | 11 (61) 33 (22) 0 (0) | **<0.0001** Ref |
| Removed manure of sows in the last 7 days | | Yes No Missing | 28 140 3 | 12 (43) 32 (23) 0 (0) | **0.02** Ref |
| Other activities with sows in the last 7 days | | Yes No Missing | 19 149 3 | 8 (42) 36 (24) 0 (0) | 0.15 Ref |
| Health care of piglets in the last 7 days | | Yes No Missing | 31 137 3 | 16 (52) 28 (20) 0 (0) | **<0.0001** Ref |
| Removed manure of piglets in the last 7 days | | Yes No Missing | 12 156 0 | 5 (42) 39 (25) 0 (0) | 0.13 Ref |
| Moved weaned piglets in the last 7 days | | Yes No Missing | 22 146 3 | 11 (50) 33 (23) 0 (0) | **<0.0001** Ref |
| Feeded weaned piglets in the last 7 days | | Yes No Missing | 15 153 3 | 6 (40) 38 (25) 0 (0) | 0.10 Ref |
| Healthcare of weaned piglets in the last 7 days | | Yes No Missing | 14 154 3 | 8 (57) 36 (23) 0 (0) | **<0.0001** Ref |
| Feeded finisher pigs in the last 7 days | | Yes No Missing | 11 157 3 | 6 (55) 38 (24) 0 (0) | **<0.0001** Ref |
| *Environment / surroundings* | |  |  |  |  |
| Lives in family with MRSA-positive pig farmer | | Yes No | 140 31 | 43 (31) 2 (6) | 0.09 Ref |
| Wet wipe samples of remote control positive for MRSA on day 0 | | Yes No Missing | 43 125 3 | 19 (44) 26 (21) 0 (0) | **0.04** Ref |
| Wet wipe samples of back door positive for MRSA on day 0 | | Yes No Missing | 49 119 3 | 20 (41) 25 (21) 0 (0) | 0.07 Ref |
| Wet wipe samples of pet positive for MRSA on day 0 | | Yes No Missing | 119 39 13 | 36 (30) 5 (13) 0 (0) | 0.16 Ref |
| Wet wipe samples of farmers chair pos on day 0 | | Yes No Missing | 94 69 8 | 33 (35) 10 (14) 0 (0) | **0.04** Ref |
| *Determinants not used for multivariate analysis (too many missings)* | | | | |  |
| Pig contact in the last 12 months | Daily Weekly Monthly <Monthly Missing | | 6 45 37 34 49 | 6 (100) 20 (44) 8 (22) 5 (15) 6 (12) | **<0.0001 0.03** 0.46 Ref 0.83 |
| Wear facemask in stables | Continuously Sometimes Never Missing | | 7 20 95 49 | 0 (0) 8 (40) 31 (33) 6 (12) | **0.01** Ref Ref |

MRSA, methicillin-resistant *Staphylococcus aureus*; MSSA, methicillin-susceptible *Staphylococcus aureus*; Ref, reference category. Bold-typed p-values were statistically significant (i.e. <0.05).

^a^ All determinants with univariate Chi-square p-values ≤0.20, prevalence >5%, number of missing observations ≤20%, and highest PR or lowest p-values when collinear (*i.e.* Spearman’s rho >0.70) were shown.
